# Effectiveness of COVID-19 mRNA Vaccines Against COVID-19–Associated Hospitalizations Among Immunocompromised Adults During SARS-CoV-2 Omicron Predominance — VISION Network, 10 States, December 2021—August 2022

**DOI:** 10.15585/mmwr.mm7142a4

**Published:** 2022-10-21

**Authors:** Amadea Britton, Peter J. Embi, Matthew E. Levy, Manjusha Gaglani, Malini B. DeSilva, Brian E. Dixon, Kristin Dascomb, Palak Patel, Kristin E. Schrader, Nicola P. Klein, Toan C. Ong, Karthik Natarajan, Emily Hartmann, Anupam B. Kharbanda, Stephanie A. Irving, Monica Dickerson, Margaret M. Dunne, Chandni Raiyani, Shaun J. Grannis, Edward Stenehjem, Ousseny Zerbo, Suchitra Rao, Jungmi Han, Chantel Sloan-Aagard, Eric P. Griggs, Zachary A. Weber, Kempapura Murthy, William F. Fadel, Nancy Grisel, Charlene McEvoy, Ned Lewis, Michelle A. Barron, Juan Nanez, Sarah E. Reese, Mufaddal Mamawala, Nimish R. Valvi, Julie Arndorfer, Kristin Goddard, Duck-Hye Yang, Bruce Fireman, Sarah W. Ball, Ruth Link-Gelles, Allison L. Naleway, Mark W. Tenforde

**Affiliations:** ^1^CDC COVID-19 Emergency Response Team; ^2^Center for Biomedical Informatics, Regenstrief Institute, Indianapolis, Indiana; ^3^School of Medicine, Indiana University, Indianapolis, Indiana; ^4^Vanderbilt University Medical Center, Nashville, Tennessee; ^5^Westat, Rockville, Maryland; ^6^Baylor Scott & White Health, Temple, Texas; ^7^School of Medicine, Texas A&M University, Bryan, Texas; ^8^HealthPartners Institute, Minneapolis, Minnesota; ^9^Richard M. Fairbanks School of Public Health, Indiana University, Indianapolis, Indiana; ^10^Division of Infectious Diseases and Clinical Epidemiology, Department of Medicine, Intermountain Healthcare, Salt Lake City, Utah; ^11^Vaccine Study Center, Division of Research, Kaiser Permanente Northern California, Oakland, California; ^12^School of Medicine, University of Colorado Anschutz Medical Campus, Aurora, Colorado; ^13^Department of Biomedical Informatics, Columbia University Irving Medical Center, New York, New York; ^14^New York-Presbyterian Hospital, New York, New York; ^15^Paso del Norte Health Information Exchange, El Paso, Texas; ^16^Children’s Minnesota, Minneapolis, Minnesota; ^17^Center for Health Research, Kaiser Permanente Northwest, Portland, Oregon; ^18^Department of Public Health, College of Life Sciences, Brigham Young University, Provo, Utah.

Persons with moderate-to-severe immunocompromising conditions might have reduced protection after COVID-19 vaccination, compared with persons without immunocompromising conditions (*1*–*3*). On August 13, 2021, the Advisory Committee on Immunization Practices (ACIP) recommended that adults with immunocompromising conditions receive an expanded primary series of 3 doses of an mRNA COVID-19 vaccine. ACIP followed with recommendations on September 23, 2021, for a fourth (booster) dose and on September 1, 2022, for a new bivalent mRNA COVID-19 vaccine booster dose, containing components of the BA.4 and BA.5 sublineages of the Omicron (B.1.1.529) variant (*4*). Data on vaccine effectiveness (VE) of monovalent COVID-19 vaccines among persons with immunocompromising conditions since the emergence of the Omicron variant in December 2021 are limited. In the multistate VISION Network,^§^ monovalent 2-, 3-, and 4-dose mRNA VE against COVID-19–related hospitalization were estimated among adults with immunocompromising conditions^¶^ hospitalized with COVID-19–like illness,** using a test-negative design comparing odds of previous vaccination among persons with a positive or negative molecular test result (case-patients and control-patients) for SARS-CoV-2 (the virus that causes COVID-19). During December 16, 2021–August 20, 2022, among SARS-CoV-2 test-positive case-patients, 1,815 (36.3%), 1,387 (27.7%), 1,552 (31.0%), and 251 (5.0%) received 0, 2, 3, and 4 mRNA COVID-19 vaccine doses, respectively. Among test-negative control-patients during this period, 6,928 (23.7%), 7,411 (25.4%), 12,734 (43.6%), and 2,142 (7.3%) received these respective doses. Overall, VE against COVID-19–related hospitalization among adults with immunocompromising conditions hospitalized for COVID-like illness during Omicron predominance was 36% ≥14 days after dose 2, 69% 7–89 days after dose 3, and 44% ≥90 days after dose 3. Restricting the analysis to later periods when Omicron sublineages BA.2/BA.2.12.1 and BA.4/BA.5 were predominant and 3-dose recipients were eligible to receive a fourth dose, VE was 32% ≥90 days after dose 3 and 43% ≥7 days after dose 4. Protection offered by vaccination among persons with immunocompromising conditions during Omicron predominance was moderate even after a 3-dose monovalent primary series or booster dose. Given the incomplete protection against hospitalization afforded by monovalent COVID-19 vaccines, persons with immunocompromising conditions might benefit from updated bivalent vaccine booster doses that target recently circulating Omicron sublineages, in line with ACIP recommendations. Further, additional protective recommendations for persons with immunocompromising conditions, including the use of prophylactic antibody therapy, early access to and use of antivirals, and enhanced nonpharmaceutical interventions such as well-fitting masks or respirators, should also be considered.

VISION Network methods to assess VE have been previously described (*3*,*5*). For this analysis, among adults aged ≥18 years, eligible medical encounters were defined as hospitalizations of patients with one or more immunocompromising conditions and a COVID-19–like illness diagnosis who underwent SARS-CoV-2 molecular testing ≤14 days before to <72 hours after the encounter date. Immunocompromising conditions were identified from electronic medical records based on *International Classification of Diseases, Ninth Revision *(ICD-9) and *International Classification of Diseases, Tenth Revision* (ICD-10) discharge diagnosis codes associated with being immunocompromised (*3*). Vaccination status was obtained from electronic health records or immunization registries. Two-dose vaccination was defined as receipt of a second dose of mRNA-1273 (Moderna) or BNT162b2 (Pfizer-BioNTech) vaccine ≥14 days before the index date;^††^ 3- and 4-dose vaccinations were defined as receipt of the most recent dose ≥7 days before the index date. Persons with no documented COVID-19 vaccine doses were considered unvaccinated. Encounters for persons who received a non-mRNA COVID-19 vaccine, only 1 dose, >4 doses, dose 2 <14 days before the index date, dose 3 or 4 <7 days before the index date, or who received doses before vaccine was recommended by ACIP were excluded.^§§^ The study period began on the date when ≥50% of sequenced specimens for each study site yielded an Omicron variant based on local surveillance data (site-specific start dates ranged from December 16 to 29, 2021) and ended August 20, 2022; start and end dates for Omicron sublineage predominance periods for BA.1 (including the original BA.1.1.529 variant and BA.1.1 and BA.1 sublineages), BA.2/BA.2.12.1, and BA.4/BA.5 were defined as the site-specific dates of ≥50% sublineage predominance^¶¶^^,^***^,^^†††^.

VE was estimated using a test-negative design, comparing the odds of being vaccinated versus unvaccinated between persons with a positive or negative SARS-CoV-2 molecular test result (case-patients and control-patients, respectively). Multivariable logistic regression models were adjusted for age, geographic region,^§§§^ calendar time, and local percentage of positive SARS-CoV-2 test results^¶¶¶^ and weighted for the inverse propensity to be vaccinated or unvaccinated**** (*5*). VE of 2- and 3-doses was estimated for the full Omicron period (all sublineages combined) and for each sublineage predominance period. VE estimates for 4 doses were restricted to a combined period including BA.2/BA.2.12.1 and BA.4/BA.5 periods because of limited 4-dose coverage among eligible persons before mid-March 2022.^††††^ VE was estimated among all persons with one or more immunocompromising condition and then separately among persons who had a single condition in one of five mutually exclusive immunocompromising condition categories: 1) solid malignancies, 2) hematologic malignancies, 3) rheumatologic or inflammatory disorders, 4) other intrinsic immune conditions or immunodeficiencies, or 5) organ or stem cell transplants. VE was also estimated among recipients of an organ or stem cell transplant without excluding those with other immunocompromising conditions and among persons with any immunocompromising condition except an organ or stem cell transplant. Estimates with nonoverlapping 95% CIs were considered significantly different. Analyses were conducted using R software (version 4.1.1; R Foundation). The study was reviewed and approved by institutional review boards at participating sites or under a reliance agreement with the institutional review board of Westat, Inc. This activity was conducted consistent with applicable federal law and CDC policy.^§§§§^

During December 16, 2021–August 20, 2022, among 34,220 eligible hospitalizations for COVID-19–like illness in adults with immunocompromising conditions (median age = 69 years; IQR = 58–78 years), 8,798 (25.7%), 14,286 (41.7%), and 2,393 (7.0%) patients had received 2, 3, and 4 COVID-19 vaccine doses, respectively, including 11,088 (32.4% of all patients included) who received dose 3 ≥90 days before the index date and were therefore eligible for a fourth dose (Table 1). VE during the full Omicron period was 36% (95% CI = 30–41) ≥14 days after dose 2, 69% (95% CI = 63–74) 7–89 days after dose 3, and 44% (95% CI = 37–49) ≥90 days after dose 3 (Table 2). When stratified by sublineage period, VE was higher ≥7 days after receipt of dose 3 during the BA.1 period (67%; median interval since vaccination = 99 days) than during the BA.2/BA.2.12.1 (32%; median interval = 172 days) and BA.4/BA.5 periods (35%; median interval = 239 days). During the combined BA2/BA.2.12.1 and BA.4/BA.5 periods, when persons with immunocompromising conditions were eligible to receive a fourth dose, VE ≥90 days after dose 3 was 32% (median interval = 196 days), and ≥7 days after dose 4 was 43% (median interval = 61 days).

**TABLE 1:** Characteristics of hospitalizations among immunocompromised* adults aged ≥18 years with COVID-19–like illness,^†^ by mRNA COVID-19 vaccination status and SARS-CoV-2 test result — VISION Network, 10 states, December 2021–August 2022

Characteristic	Total No. (column %)	mRNA COVID-19 vaccination status^§^						Positive SARS-CoV-2 test result	
		No. (row %)					SMD^¶^		
		Unvaccinated	2 doses ≥14 days earlier	3 doses 7–89 days earlier	3 doses ≥90 days earlier	4 doses ≥7 days earlier		No. (row %)	SMD^¶^
**All hospitalizations**	**34,220 (100.0)**	**8,743 (25.5)**	**8,798 (25.7)**	**3,198 (9.3)**	**11,088 (32.4)**	**2,393 (7.0)**	**NA**	**5,005 (14.6)**	**NA**
**Omicron sublineage predominance period**									
BA.1**	**15,049 (44.0)**	4,422 (29.4)	4,486 (29.8)	2,638 (17.5)	3,503 (23.3)	0 (—)	0.67	3,190 (21.2)	0.55
BA.2/BA.2.12.1**^††^**	**12,470 (36.4)**	2,807 (22.5)	2,892 (23.2)	476 (3.8)	5,172 (41.5)	1,123 (9.0)		862 (6.9)	
BA.4/BA.5**^§§^**	**6,701 (19.6)**	1,514 (22.6)	1,420 (21.2)	84 (1.3)	2,413 (36.0)	1,270 (19.0)		953 (14.2)	
**Site**									
Baylor Scott & White Health	**7,513 (22.0)**	2,722 (36.2)	2,640 (35.1)	397 (5.3)	1,639 (21.8)	115 (1.5)	0.83	1,194 (15.9)	0.17
Columbia University	**2,375 (6.9)**	650 (27.4)	646 (27.2)	248 (10.4)	737 (31.0)	94 (4.0)		355 (14.9)	
HealthPartners	**2,043 (6.0)**	337 (16.5)	353 (17.3)	259 (12.7)	834 (40.8)	260 (12.7)		251 (12.3)	
Intermountain Healthcare	**2,323 (6.8)**	607 (26.1)	539 (23.2)	268 (11.5)	776 (33.4)	133 (5.7)		483 (20.8)	
KPNC	**9,355 (27.3)**	958 (10.2)	1,810 (19.3)	1,157 (12.4)	4,079 (43.6)	1,351 (14.4)		1,253 (13.4)	
KPNW	**1,966 (5.7)**	493 (25.1)	355 (18.1)	203 (10.3)	675 (34.3)	240 (12.2)		211 (10.7)	
PHIX	**189 (0.6)**	72 (38.1)	49 (25.9)	15 (7.9)	45 (23.8)	8 (4.2)		37 (19.6)	
Regenstrief Institute	**5,132 (15.0)**	1,829 (35.6)	1,390 (27.1)	402 (7.8)	1,424 (27.7)	87 (1.7)		758 (14.8)	
University of Colorado	**3,324 (9.7)**	1,075 (32.3)	1,016 (30.6)	249 (7.5)	879 (26.4)	105 (3.2)		463 (13.9)	
**Age group, yrs**									
18–49	**4,605 (13.5)**	2,044 (44.4)	1,358 (29.5)	302 (6.6)	820 (17.8)	81 (1.8)	0.54	666 (14.5)	0.03
50–64	**8,617 (25.2)**	2,658 (30.8)	2,552 (29.6)	788 (9.1)	2,256 (26.2)	363 (4.2)		1,304 (15.1)	
65–74	**9,684 (28.3)**	2,084 (21.5)	2,372 (24.5)	956 (9.9)	3,515 (36.3)	757 (7.8)		1,373 (14.2)	
75–84	**7,885 (23.0)**	1,390 (17.6)	1,759 (22.3)	747 (9.5)	3,174 (40.3)	815 (10.3)		1,142 (14.5)	
≥85	**3,429 (10.0)**	567 (16.5)	757 (22.1)	405 (11.8)	1,323 (38.6)	377 (11.0)		520 (15.2)	
**Sex**									
Male	**16,533 (48.3)**	4,296 (26.0)	4,100 (24.8)	1,544 (9.3)	5,383 (32.6)	1,210 (7.3)	0.03	2,449 (14.8)	0.01
Female	**17,687 (51.7)**	4,447 (25.1)	4,698 (26.6)	1,654 (9.4)	5,705 (32.3)	1,183 (6.7)		2,556 (14.5)	
**Race and ethnicity**									
White, non-Hispanic	**22,318 (65.2)**	5,498 (24.6)	5,458 (24.5)	2,050 (9.2)	7,632 (34.2)	1,680 (7.5)	0.27	3,149 (14.1)	0.08
Black, non-Hispanic	**3,805 (11.1)**	1,226 (32.2)	1,118 (29.4)	364 (9.6)	966 (25.4)	131 (3.4)		642 (16.9)	
Hispanic	**4,530 (13.2)**	1,211 (26.7)	1,357 (30.0)	430 (9.5)	1,264 (27.9)	268 (5.9)		728 (16.1)	
Other,^¶¶^ non-Hispanic	**2,805 (8.2)**	489 (17.4)	671 (23.9)	318 (11.3)	1,021 (36.4)	306 (10.9)		380 (13.5)	
Unknown	**762 (2.2)**	319 (41.9)	194 (25.5)	36 (4.7)	205 (26.9)	8 (1.0)		106 (13.9)	
Documented previous SARS-CoV-2 infection***									
Yes	**4,672 (13.7)**	1,313 (28.1)	1,423 (30.5)	357 (7.6)	1,330 (28.5)	249 (5.3)	0.09	543 (11.6)	0.10
No	**29,548 (86.3)**	7,430 (25.1)	7,375 (25.0)	2,841 (9.6)	9,758 (33.0)	2,144 (7.3)		4,462 (15.1)	
**Chronic respiratory condition^†††^**									
Yes	**21,648 (63.3)**	5,419 (25.0)	5,555 (25.7)	2,073 (9.6)	7,067 (32.6)	1,534 (7.1)	0.04	3,519 (16.3)	0.18
No	**12,572 (36.7)**	3,324 (26.4)	3,243 (25.8)	1,125 (8.9)	4,021 (32.0)	859 (6.8)		1,486 (11.8)	
**Solid malignancy**									
Yes	**13,875 (40.5)**	3,234 (23.3)	3,458 (24.9)	1,290 (9.3)	4,858 (35.0)	1,035 (7.5)	0.10	1,433 (10.3)	0.29
No	**20,345 (59.5)**	5,509 (27.1)	5,340 (26.2)	1,908 (9.4)	6,230 (30.6)	1,358 (6.7)		3,572 (17.6)	
**Hematologic malignancy**									
Yes	**4,992 (14.6)**	1,086 (21.8)	1,231 (24.7)	494 (9.9)	1,765 (35.4)	416 (8.3)	0.10	789 (15.8)	0.04
No	**29,228 (85.4)**	7,657 (26.2)	7,567 (25.9)	2,704 (9.3)	9,323 (31.9)	1,977 (6.8)		4,216 (14.4)	
**Rheumatologic or inflammatory disorder**									
Yes	**8,341 (24.4)**	2,062 (24.7)	2,184 (26.2)	804 (9.6)	2,689 (32.2)	602 (7.2)	0.03	1,443 (17.3)	0.12
No	**25,879 (75.6)**	6,681 (25.8)	6,614 (25.6)	2,394 (9.3)	8,399 (32.5)	1,791 (6.9)		3,562 (13.8)	
**Other intrinsic immune condition or immunodeficiency**									
Yes	**13,183 (38.5)**	3,754 (28.5)	3,554 (27.0)	1,114 (8.5)	3,951 (30.0)	810 (6.1)	0.14	2,242 (17.0)	0.15
No	**21,037 (61.5)**	4,989 (23.7)	5,244 (24.9)	2,084 (9.9)	7,137 (33.9)	1,583 (7.5)		2,763 (13.1)	
**Organ or stem cell transplant**									
Yes	**2,951 (8.6)**	509 (17.2)	747 (25.3)	263 (8.9)	1,150 (39.0)	282 (9.6)	0.14	699 (23.7)	0.20
No	**31,269 (91.4)**	8,234 (26.3)	8,051 (25.7)	2,935 (9.4)	9,938 (31.8)	2,111 (6.8)		4,306 (13.8)	
**mRNA COVID-19 vaccination product received**									
Moderna (mRNA-1273)	**9,555 (37.5)**	NA	3,461 (36.2)	1,284 (13.4)	3,913 (41.0)	897 (9.4)	NA	1,098 (11.5)	0.11
Pfizer-BioNTech (BNT162b2)	**14,769 (58.0)**	NA	5,293 (35.8)	1,620 (11.0)	6,584 (44.6)	1,272 (8.6)		1,983 (13.4)	
Heterologous	**1,153 (4.5)**	NA	44 (3.8)	294 (25.5)	591 (51.3)	224 (19.4)		109 (9.5)	
**ICU admission**									
Yes	**7,840 (22.9)**	2,276 (29.0)	2,119 (27.0)	685 (8.7)	2,307 (29.4)	453 (5.8)	0.11	1,100 (14.0)	0.03
No	**26,380 (77.1)**	6,467 (24.5)	6,679 (25.3)	2,513 (9.5)	8,781 (33.3)	1,940 (7.4)		3,905 (14.8)	
**In-hospital death^§§§^**									
Yes	**2,741 (8.0)**	915 (33.4)	702 (25.6)	213 (7.8)	746 (27.2)	165 (6.0)	0.12	609 (22.2)	0.16
No	**31,479 (92.0)**	7,828 (24.9)	8,096 (25.7)	2,985 (9.5)	10,342 (32.9)	2,228 (7.1)		4,396 (14.0)	

**Abbreviations:** ICD-9 = *International Classification of Diseases, Ninth Revision*; ICD-10 = *International Classification of Diseases, Tenth Revision;* ICU = intensive care unit; KPNC = Kaiser Permanente Northern California; KPNW = Kaiser Permanente Northwest; NA = not applicable; PHIX = Paso del Norte Health Information Exchange; SMD = standardized mean or proportion difference.

* Immunocompromised status was defined as the presence of at least one discharge diagnosis using ICD-9 and ICD-10 diagnosis codes for solid malignancy (ICD-10 codes: C00–C80, C7A, C7B, D3A, Z51.0, and Z51.1), hematologic malignancy (ICD-10 codes: C81–C86, C88, C90–C96, D46, D61.0, D70.0, D61.2, D61.9, and D71), rheumatologic or inflammatory disorder (ICD-10 codes: D86, E85 [except E85.0], G35, J67.9, L40.54, L40.59, L93.0, L93.2, L94, M05–M08, M30, M31.3, M31.5, M32–M34, M35.3, M35.8, M35.9, M46, and T78.40), other intrinsic immune condition or immunodeficiency (ICD-10 codes: D27.9, D61.09, D72.89, D80, D81 [except D81.3], D82–D84, D89 [except D89.2], K70.3, K70.4, K72, K74.3–K74.6 [except K74.60 and K74.69], N04, and R18), or organ or stem cell transplant (ICD-10 codes: T86 [except T86.82–T86.84, T86.89, and T86.9], D47.Z1, Z48.2, Z94, and Z98.85).

**^†^** Hospitalizations with a discharge code consistent with COVID-19–like illness and molecular testing for SARS-CoV-2 ≤14 days before to <72 hours after the encounter date were included. COVID-19–like illness diagnoses included acute respiratory illness (e.g., respiratory failure or pneumonia) or related signs or symptoms (cough, fever, dyspnea, vomiting, or diarrhea) using ICD-9 and ICD-10 diagnosis codes.

^§^ mRNA COVID-19 vaccination status was defined as having received the listed number of doses of an mRNA COVID-19 vaccine within the specified range of number of days before the encounter index date, which was the date of respiratory specimen collection associated with the most recent positive or negative SARS-CoV-2 test result before the hospital admission or the admission date if testing only occurred after the admission.

^¶^ An absolute SMD >0.20 indicates a nonnegligible difference in variable distributions between hospitalizations for vaccinated versus unvaccinated patients or for patients with positive SARS-CoV-2 test results versus patients with negative SARS-CoV-2 test results. For mRNA COVID-19 vaccination status, a single SMD was calculated by averaging the absolute SMDs obtained from pairwise comparisons of each vaccinated category versus unvaccinated. Specifically, it was calculated as the average of the absolute value of the SMDs for 1) vaccinated with 2 doses ≥14 days earlier versus unvaccinated, 2) vaccinated with 3 doses 7–89 days earlier versus unvaccinated, 3) vaccinated with 3 doses ≥90 days earlier versus unvaccinated, and 4) vaccinated with 4 doses ≥7 days earlier versus unvaccinated.

** Partners contributing data on hospitalizations during dates of estimated ≥50% Omicron BA.1 predominance were in California (December 21, 2021–March 20, 2022), Colorado (December 19, 2021–March 20, 2022), Indiana (December 26, 2021–March 20, 2022), Minnesota and Wisconsin (December 25, 2021–March 21, 2022), New York (December 18, 2021–March 16, 2022), Oregon and Washington (December 24, 2021–March 23, 2022), Texas (Baylor Scott & White Health: December 16, 2021–March 18, 2022; PHIX: December 29, 2021–March 29, 2022), and Utah (December 24, 2021–March 18, 2022).

^††^ Partners contributing data on hospitalizations during dates of estimated ≥50% Omicron BA.2/BA.2.12.1 predominance were in California (March 21–June 24, 2022), Colorado (March 21–June 18, 2022), Indiana (March 21–June 18, 2022), Minnesota and Wisconsin (March 22–June 21, 2022), New York (March 17–June 28, 2022), Oregon and Washington (March 24–June 28, 2022), Texas (Baylor Scott & White Health: March 19–June 21, 2022; PHIX: March 30–June 21, 2022), and Utah (March 19–June 22, 2022).

^§§^ Partners contributing data on hospitalizations during dates of estimated ≥50% Omicron BA.4/BA.5 predominance were in California (June 25–August 20, 2022), Colorado (June 19–August 20, 2022), Indiana (June 19–August 20, 2022), Minnesota and Wisconsin (June 22–August 20, 2022), New York (June 29–August 20, 2022), Oregon and Washington (June 29–August 20, 2022), Texas (Baylor Scott & White Health: June 22–August 20, 2022; PHIX: June 22–August 20, 2022), and Utah (June 23–August 20, 2022).

^¶¶^ Other race includes American Indian or Alaska Native, Asian, Native Hawaiian or other Pacific islander, other not listed, and multiple races. These categories were combined because of small numbers.

*** Previous SARS-CoV-2 infection was defined as having a positive SARS-CoV-2 test result (molecular or antigen) documented in the electronic health record ≥15 days before the hospital admission date.

^†††^ Chronic respiratory condition was defined by corresponding discharge codes for asthma, chronic obstructive pulmonary disease, or other lung disease using ICD-9 and ICD-10 diagnosis codes.

^§§§^ In-hospital death was defined as death while hospitalized within 28 days after admission.

**TABLE 2:** Vaccine effectiveness* of 2-, 3-, and 4-dose mRNA COVID-19 vaccination against COVID-19–associated^†^ hospitalizations among immunocompromised^§^ adults aged ≥18 years, by Omicron (and Omicron sublineage) predominance period^¶^ and mRNA COVID-19 vaccination status** — VISION Network, 10 states, December 2021–August 2022

Omicron predominance period/vaccination status	Total	SARS-CoV-2 positive test result, no. (%)	Median interval since last dose, days (IQR)	VE % (95% CI)
**Omicron predominance period**				
Unvaccinated (Ref)	**8,743**	1,815 (20.8)	NA	NA
2 doses (≥14 days earlier)	**8,798**	1,387 (15.8)	316 (250–387)	36 (30–41)
3 doses (≥7 days earlier)	**14,286**	1,552 (10.9)	147 (96–202)	57 (53–61)
3 doses (7–89 days earlier)	**3,198**	335 (10.5)	59 (38–76)	69 (63–74)
3 doses (≥90 days earlier)	**11,088**	1,217 (11.0)	169 (131–218)	44 (37–49)
**BA.1 sublineage predominance^††^**				
Unvaccinated (Ref)	**4,422**	1,373 (31.1)	NA	NA
2 doses (≥14 days earlier)	**4,486**	1,008 (22.5)	283 (222–321)	40 (34–46)
3 doses (≥7 days earlier)	**6,141**	809 (13.2)	99 (65–133)	67 (63–71)
3 doses (7–89 days earlier)	**2,638**	302 (11.4)	59 (38–75)	75 (71–79)
3 doses (≥90 days earlier)	**3,503**	507 (14.5)	128 (109–152)	49 (41–7)
**BA.2/BA.2.12.1 sublineage predominance^§§^**				
Unvaccinated (Ref)	**2,807**	190 (6.8)	NA	NA
2 doses (≥14 days earlier)	**2,892**	204 (7.1)	371 (286–414)	7 (–16–25)
3 doses (≥7 days earlier)	**5,648**	372 (6.6)	172 (134–210)	32 (16–46)
3 doses (7–89 days earlier)	—^¶¶^	—	—	—
3 doses (≥90 days earlier)	**5,172**	351 (6.8)	179 (145–214)	32 (15–45)
BA.4/BA.5 sublineage predominance***				
Unvaccinated (Ref)	**1,514**	252 (16.6)	NA	NA
2 doses (≥14 days earlier)	**1,420**	175 (12.3)	445 (336–488)	38 (23–50)
3 doses (≥7 days earlier)	**2,497**	371 (14.9)	239 (199–276)	35 (21–47)
3 doses (7–89 days earlier)	—	—	—	—
3 doses (≥90 days earlier)	**2,413**	359 (14.9)	241 (204–278)	36 (22–47)
**BA.2/BA.2.12.1/BA.4/BA.5 sublineage predominance^†††^**				
Unvaccinated (Ref)	**4,321**	442 (10.2)	NA	NA
2 doses (≥14 days earlier)	**4,312**	379 (8.8)	386 (305–441)	22 (10–33)
3 doses (≥7 days earlier)	**8,145**	743 (9.1)	190 (147–234)	33 (22–42)
3 doses (7–89 days earlier)	—	—	—	—
3 doses (≥90 days earlier)	**7,585**	710 (9.4)	196 (156–238)	32 (21–42)
4 doses (≥7 days earlier)	**2,393**	251 (10.5)	61 (34–91)	43 (27–56)

**Abbreviations**: ICD-9 = *International Classification of Diseases, Ninth Revision*; ICD-10 = *International Classification of Diseases, Tenth Revision*; NA = not applicable; PHIX = Paso del Norte Health Information Exchange; Ref = referent group; VE = vaccine effectiveness.

* VE was calculated as ([1 − odds ratio] x 100%), estimated using a test-negative design, adjusted for age, geographic region, calendar time (days since January 1, 2021), and local virus circulation (percentage of SARS-CoV-2–positive results from testing within the counties surrounding the facility on the date of the encounter) and weighted for inverse propensity to be vaccinated or unvaccinated (calculated separately for each VE estimate). Generalized boosted regression trees were used to estimate the propensity to be vaccinated based on sociodemographic characteristics, underlying medical conditions, and facility characteristics.

^†^ Hospitalizations with a discharge code consistent with COVID-19–like illness and molecular testing for SARS-CoV-2 ≤14 days before to <72 hours after the encounter date were included. COVID-19–like illness diagnoses included acute respiratory illness (e.g., respiratory failure or pneumonia) or related signs or symptoms (cough, fever, dyspnea, vomiting, or diarrhea) using ICD-9 and ICD-10 diagnosis codes.

^§^ Immunocompromised status was defined as the presence of at least one discharge diagnosis using ICD-9 and ICD-10 diagnosis codes for solid malignancy (ICD-10 codes: C00–C80, C7A, C7B, D3A, Z51.0, and Z51.1), hematologic malignancy (ICD-10 codes: C81–C86, C88, C90–C96, D46, D61.0, D70.0, D61.2, D61.9, and D71), rheumatologic or inflammatory disorder (ICD-10 codes: D86, E85 [except E85.0], G35, J67.9, L40.54, L40.59, L93.0, L93.2, L94, M05–M08, M30, M31.3, M31.5, M32–M34, M35.3, M35.8, M35.9, M46, and T78.40), other intrinsic immune condition or immunodeficiency (ICD-10 codes: D27.9, D61.09, D72.89, D80, D81 [except D81.3], D82–D84, D89 [except D89.2], K70.3, K70.4, K72, K74.3–K74.6 [except K74.60 and K74.69], N04, and R18), or organ or stem cell transplant (ICD-10 codes: T86 [except T86.82–T86.84, T86.89, and T86.9], D47.Z1, Z48.2, Z94, and Z98.85).

^¶^ Based on ≥50% of sequenced specimens yielding a specific Omicron sublineage.

** mRNA COVID-19 vaccination status was defined as having received the listed number of doses of an mRNA COVID-19 vaccine within the specified range of number of days before the encounter index date, which was the date of respiratory specimen collection associated with the most recent positive or negative SARS-CoV-2 test result before the hospital admission or the admission date if testing only occurred after the admission.

^††^ Partners contributing data on hospitalizations during dates of estimated ≥50% Omicron BA.1 predominance were in California (December 21, 2021–March 20, 2022), Colorado (December 19, 2021–March 20, 2022), Indiana (December 26, 2021–March 20, 2022), Minnesota and Wisconsin (December 25, 2021–March 21, 2022), New York (December 18, 2021–March 16, 2022), Oregon and Washington (December 24, 2021–March 23, 2022), Texas (Baylor Scott & White Health: December 16, 2021–March 18, 2022; PHIX: December 29, 2021–March 29, 2022), and Utah (December 24, 2021–March 18, 2022).

^§§^ Partners contributing data on hospitalizations during dates of estimated ≥50% Omicron BA.2/BA.2.12.1 predominance were in California (March 21–June 24, 2022), Colorado (March 21–June 18, 2022), Indiana (March 21–June 18, 2022), Minnesota and Wisconsin (March 22–June 21, 2022), New York (March 17–June 28, 2022), Oregon and Washington (March 24–June 28, 2022), Texas (Baylor Scott & White Health: March 19–June 21, 2022; PHIX: March 30–June 21, 2022), and Utah (March 19–June 22, 2022).

^¶¶^ Dashes indicate that estimated VE had a CI width ≥50%. Estimates with CI widths ≥50% are not shown here due to imprecision. The associated data (total number of tests, number of SARS-CoV-2 positive tests, and median interval since last dose) are also omitted.

*** Partners contributing data on hospitalizations during dates of estimated ≥50% Omicron BA.4/BA.5 predominance were in California (June 25–August 20, 2022), Colorado (June 19–August 20, 2022), Indiana (June 19–August 20, 2022), Minnesota and Wisconsin (June 22–August 20, 2022), New York (June 29–August 20, 2022), Oregon and Washington (June 29–August 20, 2022), Texas (Baylor Scott & White Health: June 22–August 20, 2022; PHIX: June 22–August 20, 2022), and Utah (June 23–August 20, 2022).

^†††^ Partners contributing data on hospitalizations during dates of estimated ≥50% Omicron BA.2/BA.2.12.1/BA.4/BA.5 predominance were in California (March 21–August 20, 2022), Colorado (March 21–August 20, 2022), Indiana (March 21–August 20, 2022), Minnesota and Wisconsin (March 22–August 20, 2022), New York (March 17–August 20, 2022), Oregon and Washington (March 24–August 20, 2022), Texas (Baylor Scott & White Health: March 19–August 20, 2022; PHIX: March 30–August 20, 2022), and Utah (March 19–August 20, 2022).

VE ≥7 days after receipt of dose 3 varied by immunocompromising condition, ranging from 43% among persons with an organ or stem cell transplant (with or without another condition) to 70% among those with a solid malignancy only (Table 3).

**TABLE 3:** Vaccine effectiveness* of 2- and 3-dose mRNA COVID-19 vaccination against COVID-19–associated^†^ hospitalization among immunocompromised^§^ adults aged ≥18 years by immunocompromising condition category and mRNA COVID-19 vaccination status,^¶^ during period of Omicron predominance** — VISION Network, 10 states, December 2021–August 2022

Immunocompromising condition	Total	SARS-CoV-2 positive test result, no. (%)	Median interval since last dose, days (IQR)	VE % (95% CI)
**Solid malignancy only**				
Unvaccinated (Ref)	**2,467**	411 (16.7)	NA	NA
2 doses (≥14 days earlier)	**2,574**	282 (11.0)	322 (257–390)	47 (36–55)
3 doses (≥7 days earlier)	**4,523**	296 (6.5)	148 (96–203)	70 (64–76)
3 doses (7–89 days earlier)	**991**	55 (5.5)	57 (37–75)	81 (72–87)
3 doses (≥90 days earlier)	**3,532**	241 (6.8)	171 (131–219)	61 (52–69)
**Hematologic malignancy only**				
Unvaccinated (Ref)	**562**	117 (20.8)	NA	NA
2 doses (≥14 days earlier)	—^††^	—	—	—
3 doses (≥7 days earlier)	**1,209**	162 (13.4)	147 (94–204)	58 (40–70)
3 doses (7–89 days earlier)^††^	—	—	—	—
3 doses (≥90 days earlier)	**924**	104 (11.3)	171 (131–219)	63 (45–75)
**Rheumatologic or inflammatory disorder only**				
Unvaccinated (Ref)	**1,549**	378 (24.4)	NA	NA
2 doses (≥14 days earlier)	**1,528**	281 (18.4)	321 (249–394)	38 (24–49)
3 doses (≥7 days earlier)	**2,395**	253 (10.6)	141 (90–195)	61 (51–69)
3 doses (7–89 days earlier)	**599**	57 (9.5)	61 (38–76)	76 (63–84)
3 doses (≥90 days earlier)	**1,796**	196 (10.9)	166 (129–212)	48 (34–60)
**Other intrinsic immune condition or immunodeficiency only**				
Unvaccinated (Ref)	**2,334**	465 (19.9)	NA	NA
2 doses (≥14 days earlier)	**1,852**	279 (15.1)	304 (239–375)	40 (28–51)
3 doses (≥7 days earlier)	**2,222**	210 (9.4)	140 (87–196)	64 (54–72)
3 doses (7–89 days earlier)	**576**	46 (8.0)	59 (37–76)	76 (62–85)
3 doses (≥90 days earlier)	**1,646**	164 (10.0)	168 (129–215)	45 (27–58)
**Organ or stem cell transplant only**				
Unvaccinated (Ref)	**151**	47 (31.1)	NA	NA
2 doses (≥14 days earlier)	—	—	—	—
3 doses (≥7 days earlier)	—	—	—	—
3 doses (7–89 days earlier)	—	—	—	—
3 doses (≥90 days earlier)	—	—	—	—
**Organ or stem cell transplant (not mutually exclusive of other conditions)** ^§§^				
Unvaccinated (Ref)	**509**	151 (29.7)	NA	NA
2 doses (≥14 days earlier)	**747**	178 (23.8)	310 (248–378)	40 (17–56)
3 doses (≥7 days earlier)	**1,413**	326 (23.1)	153 (107–210)	43 (22–58)
3 doses (7–89 days earlier)	—	—	—	—
3 doses (≥90 days earlier)	**1,150**	265 (23.0)	170 (134–223)	30 (4–49)
**Any immunocompromising condition, except organ or stem cell transplant** ^¶¶^				
Unvaccinated (Ref)	**8,234**	1,664 (20.2)	NA	NA
2 doses (≥14 days earlier)	**8,051**	1,209 (15.0)	317 (250–387)	37 (31–42)
3 doses (≥7 days earlier)	**12,873**	1,226 (9.5)	146 (95–201)	60 (56–64)
3 doses (7–89 days earlier)	**2,935**	274 (9.3)	60 (39–76)	70 (64–75)
3 doses (≥90 days earlier)	**9,938**	952 (9.6)	169 (130–217)	47 (41–53)

**Abbreviations**: ICD-9 = *International Classification of Diseases, Ninth Revision*; ICD-10 = *International Classification of Diseases, Tenth Revision*; NA = not applicable; Ref = referent group; VE = vaccine effectiveness.

* VE was calculated as ([1 − odds ratio] x 100%), estimated using a test-negative design, adjusted for age, geographic region, calendar time (days since January 1, 2021), and local virus circulation (percentage of SARS-CoV-2–positive results from testing within the counties surrounding the facility on the date of the encounter) and weighted for inverse propensity to be vaccinated or unvaccinated (calculated separately for each VE estimate). Generalized boosted regression trees were used to estimate the propensity to be vaccinated based on sociodemographic characteristics, underlying medical conditions, and facility characteristics.

^†^ Hospitalizations with a discharge code consistent with COVID-19–like illness and molecular testing for SARS-CoV-2 ≤14 days before to <72 hours after the encounter date were included. COVID-19–like illness diagnoses included acute respiratory illness (e.g., respiratory failure or pneumonia) or related signs or symptoms (cough, fever, dyspnea, vomiting, or diarrhea) using ICD-9 and ICD-10 diagnosis codes.

^§^ Immunocompromised status was defined as the presence of at least one discharge diagnosis using ICD-9 and ICD-10 diagnosis codes (ICD-10 codes: C00–C80, C7A, C7B, D3A, Z51.0, and Z51.1), hematologic malignancy (ICD-10 codes: C81–C86, C88, C90–C96, D46, D61.0, D70.0, D61.2, D61.9, and D71), rheumatologic or inflammatory disorder (ICD-10 codes: D86, E85 [except E85.0], G35, J67.9, L40.54, L40.59, L93.0, L93.2, L94, M05–M08, M30, M31.3, M31.5, M32–M34, M35.3, M35.8, M35.9, M46, and T78.40), other intrinsic immune condition or immunodeficiency (ICD-10 codes: D27.9, D61.09, D72.89, D80, D81 [except D81.3], D82–D84, D89 [except D89.2], K70.3, K70.4, K72, K74.3–K74.6 [except K74.60 and K74.69], N04, and R18), or organ or stem cell transplant (ICD-10 codes: T86 [except T86.82–T86.84, T86.89, and T86.9], D47.Z1, Z48.2, Z94, and Z98.85).

^¶^ mRNA COVID-19 vaccination status was defined as having received the listed number of doses of an mRNA COVID-19 vaccine within the specified range of number of days before the encounter index date, which was the date of respiratory specimen collection associated with the most recent positive or negative SARS-CoV-2 test result before the hospital admission or the admission date if testing only occurred after the admission.

** Based on ≥50% of sequenced specimens yielding an Omicron variant or sublineage.

^††^ Dashes indicated that estimated VE had a CI width ≥50%. Estimates with CI widths ≥50% are not shown here due to imprecision. The associated data (total number of tests, number of SARS-CoV-2 positive tests, and median interval since last dose) are also omitted.

^§§^ Category includes persons with at least organ or stem cell transplant, but these categories are not mutually exclusive (i.e., persons might have one or more additional immunocompromising conditions).

^¶¶^ Category includes persons with one or more immunocompromising conditions: solid malignancy, hematologic malignancy, rheumatologic or inflammatory disorder, and other intrinsic immune condition or immunodeficiency; all persons with organ or stem cell transplant were excluded.

## Discussion

In this multistate analysis of over 34,000 hospitalizations for COVID-19–like illness among adults with immunocompromising conditions, 2 doses of monovalent mRNA COVID-19 vaccine were 36% effective against COVID-19–associated hospitalization during a period of Omicron variant predominance. VE increased to 67% with the addition of a third dose of monovalent vaccine during BA.1 predominance but declined during the combined BA.2/BA.2.12.1 and BA.4/BA.5 periods to 32% ≥90 days after dose 3 and 43% ≥7 days after a monovalent fourth dose. These results suggest that monovalent COVID-19 vaccination among persons with immunocompromising conditions conferred moderate protection against COVID-19–associated hospitalization during Omicron circulation, with lower protection during BA.2/BA.2.12.1 and BA.4/BA.5 sublineage predominance periods.

Although protection increased after receipt of a third monovalent vaccine dose (compared with 2 doses), estimated 3-dose VE was lower in this study than in other similar studies among immunocompetent persons during Omicron predominance, including recent VISION Network analyses (*6*,*7*). Consistent with previous studies restricted to persons with immunocompromising conditions, VE in this study was lower among persons with certain immunocompromising conditions that might be associated with being more severely immunocompromised, particularly solid organ or stem cell transplant recipients.

Estimated VE among persons with immunocompromising conditions during Omicron predominance was lower than VE in comparable studies during Delta variant predominance (*2*). Protection was also lower during Omicron BA.2/BA.2.12.1 and BA.4/BA.5 than during BA.1 predominance, although the median interval since receipt of last vaccine dose was lower during BA.1, and waning effectiveness over time might have also contributed to the lower VE observed during these later sublineage periods. In either case, these findings suggest that the newly authorized bivalent booster vaccines, which target BA.4/BA.5 might offer additional benefit to persons with immunocompromising conditions (*8*).

Given the moderate protection observed even after monovalent booster doses, persons with immunocompromising conditions might also benefit from other recommended protective measures including preexposure prophylaxis with the antibody treatment tixagevimab/cilgavimab (Evusheld),^¶¶¶¶^ which was authorized in December 2021 for persons with moderate-to-severe immunocompromising conditions and was associated with a reduction in risk for both symptomatic and severe COVID-19 in clinical trials (*9*). However, recent in vitro data suggest protection against emerging Omicron sublineages might be reduced and additional clinical data are needed (*10*).

The findings in this report are subject to at least four limitations. First, immunocompromising conditions were based on discharge diagnosis codes and a range of immune suppression is associated with each code. Second, residual confounding in VE models is possible. For example, history of previous infection could not be accurately ascertained, but might have differed between vaccinated and unvaccinated persons, which could affect VE estimates. Third, data on the use of outpatient treatments such as nirmatelvir/ritonavir (Paxlovid) or prophylaxis with Evusheld were not available. Finally, SARS-CoV-2 genomic sequencing data were unavailable for individual encounters, and date of testing was used to assign likely sublineage ecologically.

Persons with immunocompromising conditions have been disproportionately affected by the COVID-19 pandemic. Whereas monovalent vaccination remains moderately protective in persons with immunocompromising conditions, VE has decreased compared with that during pre-Omicron periods, most notably during recent Omicron sublineage predominance periods, despite expanded dosing recommendations. Given the incomplete protection against hospitalization afforded by monovalent COVID-19 vaccines, persons with immunocompromising conditions might benefit from updated bivalent boosters that target BA.4/BA.5 sublineages. In addition, other protective measures recommended for persons with immunocompromising conditions, including prophylactic antibody treatments, early access to and use of antivirals, and nonpharmaceutical interventions, such as the use of well-fitting masks or respirators, should also be considered. Further study of VE of updated vaccines in persons with immunocompromising conditions is warranted.

**Summary**

**What is already known about this topic?**

COVID-19 vaccine effectiveness (VE) data among immunocompromised persons during SARS-CoV-2 Omicron variant predominance are limited.

**What is added by this report?**

Among immunocompromised adults hospitalized with a COVID-like illness, 2-dose monovalent mRNA COVID-19 vaccine VE against COVID-19–associated hospitalization during Omicron predominance was 36%. VE was 67% ≥7 days after a third dose during BA.1 predominance but declined during BA.2/BA.2.12.1 and BA.4/BA.5 predominance to 32% ≥90 days after dose 3 and 43% ≥7 days after dose 4.

**What are the implications for public health practice?**

Monovalent COVID-19 vaccine protection among persons with immunocompromising conditions during Omicron predominance was moderate after a 3-dose primary series or booster dose. Persons with immunocompromising conditions might benefit from updated bivalent boosters that target circulating BA.4/BA.5 sublineages.

## Footnotes

* These authors contributed equally to this report.

† These senior authors contributed equally to this report.

§ VISION Network includes partner sites in California (Kaiser Permanente Northern California), Colorado (University of Colorado), Indiana (Regenstrief Institute), Minnesota (HealthPartners), New York (Columbia University Irving Medical Center), Oregon (Kaiser Permanente Northwest), Texas (Baylor Scott & White Health and Paso Del Norte Health Information Exchange), Utah (Intermountain Healthcare), Washington (Kaiser Permanente Northwest), and Wisconsin (HealthPartners).

¶ Immunocompromised status was defined as the presence of at least one discharge diagnosis, using *International Classification of Diseases, Ninth Revision *(ICD-9) and *International Classification of Diseases, Tenth Revision* (ICD-10) diagnosis codes for solid malignancy (C00–C80, C7A, C7B, D3A, Z51.0, and Z51.1), hematologic malignancy (C81–C86, C88, C90–C96, D46, D61.0, D70.0, D61.2, D61.9, and D71), rheumatologic or inflammatory disorder (D86, E85 [except E85.0], G35, J67.9, L40.54, L40.59, L93.0, L93.2, L94, M05–M08, M30, M31.3, M31.5, M32–M34, M35.3, M35.8, M35.9, M46, and T78.40), other intrinsic immune condition or immunodeficiency (D27.9, D61.09, D72.89, D80, D81 [except D81.3], D82–D84, D89 [except D89.2], K70.3, K70.4, K72, K74.3–K74.6 [except K74.60 and K74.69], N04, and R18), or organ or stem cell transplant (T86 [except T86.82–T86.84, T86.89, and T86.9], D47.Z1, Z48.2, Z94, and Z98.85).

** Hospitalizations with a discharge diagnosis code consistent with COVID-19–like illness were included. COVID-19–like illness diagnoses included acute respiratory illness (e.g., respiratory failure or pneumonia) or related signs or symptoms (e.g., cough, fever, dyspnea, vomiting, or diarrhea) using ICD-9 and ICD-10 diagnosis codes.

†† The index date for each encounter was defined as either the date of collection of a respiratory specimen associated with the most recent positive or negative SARS-CoV-2 test result before the hospital admission or the admission date (if testing occurred only after the admission).

§§ Encounters among persons who received dose 3 before recommended by ACIP (August 13, 2021) or doses outside recommended dosing intervals (<28 days between doses 2 and 3 or <90 days between doses 3 and 4) were excluded. A fifth dose (second booster) was recommended for persons with immunocompromising conditions on March 29, 2022, ≥4 months after their fourth dose. However, only eight of 223 (3.6%) potentially eligible persons during the study period had received a fifth dose ≥7 days before the index date; because of the limited sample size these persons were excluded from analyses.

¶¶ Partners contributing data on hospitalizations during dates of estimated ≥50% Omicron BA.1 predominance were in California (December 21, 2021–March 20, 2022), Colorado (December 19, 2021–March 20, 2022), Indiana (December 26, 2021–March 20, 2022), Minnesota and Wisconsin (December 25, 2021–March 21, 2022), New York (December 18, 2021–March 16, 2022), Oregon and Washington (December 24, 2021–March 23, 2022), Texas (Baylor Scott & White Health: December 16, 2021–March 18, 2022; Paso Del Norte Health Information Exchange: December 29, 2021–March 29, 2022), and Utah (December 24, 2021–March 18, 2022).

*** Partners contributing data on hospitalizations during dates of estimated ≥50% Omicron BA.2/BA.2.12.1 predominance were in California (March 21–June 24, 2022), Colorado (March 21–June 18, 2022), Indiana (March 21–June 18, 2022), Minnesota and Wisconsin (March 22–June 21, 2022), New York (March 17–June 28, 2022), Oregon and Washington (March 24–June 28, 2022), Texas (Baylor Scott & White Health: March 19–June 21, 2022; Paso Del Norte Health Information Exchange: March 30–June 21, 2022), and Utah (March 19–June 22, 2022).

††† Partners contributing data on hospitalizations during dates of estimated ≥50% Omicron BA.4/BA.5 predominance were in California (June 25–August 20, 2022), Colorado (June 19–August 20, 2022), Indiana (June 19–August 20, 2022), Minnesota and Wisconsin (June 22–August 20, 2022), New York (June 29–August 20, 2022), Oregon and Washington (June 29–August 20, 2022), Texas (Baylor Scott & White Health: June 22–August 20, 2022; Paso Del Norte Health Information Exchange: June 22–August 20, 2022), and Utah (June 23–August 20, 2022).

§§§ VISION Network site partners categorized their medical facilities into a total of 43 geographic subregions based on locations of included facilities.

¶¶¶ Local SARS-CoV-2 circulation on the day of each medical visit was defined as percentage of positive local test results reported in the U.S. Department of Health and Human Services (HHS) Protect database; data present in HHS Protect are representative of diagnostic specimens being tested and reflects the majority of, but not all, laboratory-based COVID-19 testing being conducted in the United States.

**** Covariates considered for inclusion in propensity score models and evaluated for imbalances after inverse propensity-to-be-vaccinated weighting have been previously published. An absolute standardized mean or proportion difference (SMD) >0.20 indicated a nonnegligible difference in variable distributions among events for vaccinated versus unvaccinated patients. All covariates with SMD >0.20 after weighting were also included in the multivariable logistic regression model for the respective VE estimate to minimize residual confounding.

†††† The initial recommendation for a third vaccine dose in immunocompromised persons was made on August 13, 2021; a fourth dose was recommended on September 23, 2021. The initial recommended interval between doses 3 and 4 was ≥6 months, but this was shortened to ≥5 months on January 4, 2022, and then to 3 months on February 11, 2022. Persons who received their additional primary series dose after the August 2021 recommendation and were in the initial 6-month interval would have first been eligible for a fourth dose in late February 2022. As a result, very few persons received a fourth vaccine dose before March 2022. https://www.cdc.gov/vaccines/covid-19/clinical-considerations/interim-considerations-us-appendix.html

§§§§ 45 C.F.R. part 46.102(l)(2), 21 C.F.R. part 56; 42 U.S.C. Sect. 241(d); 5 U.S.C. Sect. 552a; 44 U.S.C. Sect. 3501 et seq.

¶¶¶¶
https://www.fda.gov/drugs/drug-safety-and-availability/fda-releases-important-information-about-risk-covid-19-due-certain-variants-not-neutralized-evusheld
